# STAT3 Activity and Function in Cancer: Modulation by STAT5 and miR-146b

**DOI:** 10.3390/cancers6020958

**Published:** 2014-04-23

**Authors:** Sarah R. Walker, Michael Xiang, David A. Frank

**Affiliations:** Department of Medical Oncology, Dana-Farber Cancer Institute, and Departments of Medicine, Brigham and Women’s Hospital and Harvard Medical School, 450 Brookline Avenue, Boston, MA 02215, USA; E-Mails: sarah_walker@dfci.harvard.edu (S.R.W.); mxiang@mit.edu (M.X.)

**Keywords:** signal transduction, gene transcription, molecular oncology

## Abstract

The transcription factor STAT3 regulates genes that control critical cellular processes such as proliferation, survival, pluripotency, and motility. Thus, under physiological conditions, the transcriptional function of STAT3 is tightly regulated as one part of a complex signaling matrix. When these processes are subverted through mutation or epigenetic events, STAT3 becomes highly active and drives elevated expression of genes underlying these phenotypes, leading to malignant cellular behavior. However, even in the presence of activated STAT3, other cellular modulators can have a major impact on the biological properties of a cancer cell, which is reflected in the clinical behavior of a tumor. Recent evidence has suggested that two such key modulators are the activation status of other STAT family members, particularly STAT5, and the expression of STAT3-regulated genes that are part of negative feedback circuits, including microRNAs such as miR-146b. With attention to these newly emerging areas, we will gain greater insight into the consequence of STAT3 activation in the biology of human cancers. In addition, understanding these subtleties of STAT3 signaling in cancer pathogenesis will allow the development of more rational molecular approaches to cancer therapy.

## 1. Introduction

Signal transducers and activators of transcription (STATs) are a family of transcription factors that regulate a range of important cellular functions [1]. Under basal conditions, STATs are found inactive in the cytoplasm. Upon activation by tyrosine phosphorylation, STATs dimerize, translocate to the nucleus, bind to DNA, and regulate transcription of target genes. The STAT family is comprised of seven members, including the highly homologous STAT5a and STAT5b. The family members show diverse expression in cells and tissues, respond to distinct stimuli, and regulate partially overlapping subsets of target genes [2].

**Figure 1:**
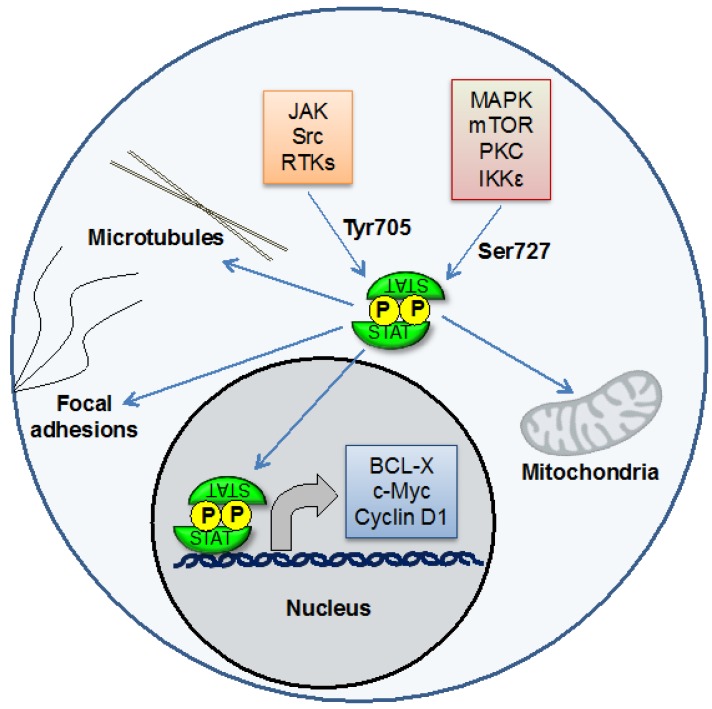
STAT3 is a transcription factor that can be activated by phosphorylation on Tyr705 by Jaks and other kinases. STAT3 then regulates the expression of genes controlling processes including proliferation, survival, invasion, and angiogenesis. STAT3 can also be phosphorylated on Ser727. This is important for its effects on mitochondrial function. STAT3 may also have effects at other cellular sites, including microtubules and focal adhesions.

STAT3, which was initially identified as a mediator of the inflammation-associated acute phase response, has been of particular interest in cancer biology [3]. Although largely studied for its role as a transcription factor, recent evidence has suggested that STAT3 can also function at other cellular sites including mitochondria, in which it modulates bioenergetics and affects cellular function independent of its nuclear role [4,5] (Figure 1). The target genes for STAT3 include those that regulate proliferation (such as cyclin D1), survival (such as Mcl-1 and Bcl-xl), pluripotency (such as KLF4), and angiogenesis and invasion (such as VEGF and several matrix metalloproteinases). Under physiological conditions, STAT3 is activated only transiently, reflecting the critical functions of its target genes. In many types of cancer, by contrast, STAT3 is activated constitutively [6]. This can result from mutated or inappropriately activated kinases, loss of negative regulators, or some combination. Thus early research on the role of STAT3 in cancer seemed to clearly indicate that constitutive activation of this protein is a direct driver of cancer pathogenesis.

Subsequently, a number of well-performed studies have suggested that the activation of STAT3 might not be uniformly associated with increased malignancy, and that in some circumstances STAT3 might act as a functional tumor suppressor or its activation may be associated with an improved prognosis [7,8,9,10]. Thus, dissecting these dichotomous roles is an important question in cancer biology.

STAT3 is activated commonly (that is, in more than half of all patients) in a wide spectrum of human cancers, including cancers of the breast, prostate, ovary, and pancreas [11,12,13]. Thus, it is difficult to make the case that it is acting as a classical tumor suppressor in any of these diseases. Quite the contrary, the fact that both upstream regulators (including kinases, receptors, and autocrine factors) and down regulators (such as SOCS proteins and phosphatases) are commonly altered to lead to STAT3 activation suggests that this is an important event in cancer pathogenesis which provides a selective advantage. However, it is clear that the consequence of STAT3 activation may be quite distinct depending on other signaling events occurring in a cell. This can include both parallel signaling events, such as the simultaneous activation of another member of the STAT family or the responsiveness of genes that can be regulated by STAT3. Recent evidence suggests that both such mechanisms may be operative in human cancer.

## 2. Co-Activation of STAT3 and STAT5 in Breast Cancer: Modulation of Gene Expression and Biology by a STAT Family Member

Although STAT3 is activated inappropriately in a majority of breast cancers, an understanding of the physiological roles of STAT transcription factor in mammary epithelium actually began with STAT5. Originally identified as “mammary gland factor”, STAT5 mediates the effect of prolactin in stimulating milk production in mammary epithelium [14,15,16]. Thus, STAT5 promotes both survival and differentiation of mammary epithelium, and its target genes include components of milk including β-casein and whey acidic protein. Physiologically, STAT5 is activated late in pregnancy and during lactation. STAT3, by contrast, is activated during the involution phase, when remodeling of the mammary gland occurs [17,18]. It seems somewhat paradoxical that STAT3, which regulates genes promoting proliferation and survival, is associated with a phase of mammary function characterized by widespread apoptosis. However, inappropriate activation of STAT3 is a common event in a broad spectrum of human epithelial cancers, and there is evidence that both STAT5 and STAT3 can become activated in breast cancers.

A number of studies have examined the activation state of STAT3 in primary breast cancers. Using a variety of approaches, including biochemical techniques such as immunoblots and electrophoretic mobility shift assays (EMSAs) as well as immunohistochemistry (IHC), a number of investigators have found evidence of constitutive activation of STAT3 in breast cancer cells but not in normal mammary tissue [11]. Similar approaches have provided evidence for constitutive activation of STAT5 in breast cancer, as well [19]. The phosphorylation of STAT5 in mammary epithelium is driven almost completely by prolactin, and prolactin promotes survival and proliferation of mammary epithelium, two key properties of breast cancer. This raised the possibility that elevated circulating levels of prolactin could be a risk factor for breast cancer. Given the long latency between exposure of potential pro-carcinogenic stimuli and the appearance of a clinically detectable tumor, the use of large longitudinal epidemiologic studies provided the best opportunity to assess a potential association. In fact, evidence derived from the Nurses Health Study did provide evidence for an association between elevated prolactin levels and breast cancers expressing the estrogen and progesterone receptor [20].

These findings then raised the question of whether an individual breast cancer could display activation of both STAT3 and STAT5, or whether these were mutually exclusive events. To address this question, tissue microarrays were stained with antibodies that could recognize the activated, tyrosine-phosphorylated form of either STAT3 or STAT5 [19]. The first key observation in these invasive ductal carcinomas, the most common type of breast cancer, was that in tumors that stained positive with either antibody, the staining was largely confined to the tumor cells and not surrounding stroma, indicating that STAT activation was a property of the malignant cells themselves. Furthermore, the staining was most intense in the nucleus, where the activated STAT exerts its transcriptional effects, indicating that it likely reflected canonical STAT function. Finally, in “positive” tumors, a majority of nuclei showed staining, suggesting that the presence of activated STAT3 or STAT5 in a given cancer specimen was an intrinsic property of that tumor, and did not merely reflect the properties of a minor sub-population.

**Figure 2:**
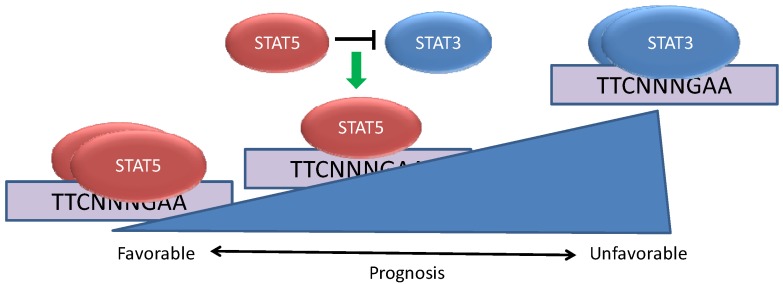
STAT5 can modulate the effects of STAT3 in breast cancer. Breast cancers can be characterized by the constitutive activation of STAT3, STAT5, or both STATs concurrently. When STAT5 is activated in conjunction with STAT3, STAT5 plays a dominant role in binding to a subset of genomic STAT binding sites and altering STAT3-dependent gene expression. The co-activation of STAT5 with STAT3 is associated with a more favorable cancer phenotype and prognosis than tumors in which STAT3 is activated alone.

A number of investigators had found that a majority of primary breast cancers display STAT3 activation. However, when analysis of both STAT3 and STAT5 activation was performed on the same tumors, it became clear that approximately 40% of the tumors with STAT3 activation also showed activation of STAT5 [19]. This raised the question of whether tumors with activated STAT3 have different biological properties if STAT5 is activated as well. Analysis of the pathological characteristics of the tumors from which these tissue microarrays were generated revealed that cancers displaying activation of both STAT3 and STAT5 were more likely to be more differentiated, low-grade tumors. In addition, they were more likely to express the estrogen receptor, and less likely to have spread to axillary lymph nodes. All of these properties of tumors with activation of both STAT3 and STAT5 reflected a more favorable clinical outcome than tumors displaying activation of STAT3 alone. In fact, patient survival was significantly prolonged in the patients whose tumors displayed gene expression signatures consistent with co-activation of STAT3 and STAT5 rather than STAT3 activation alone [21] (Figure 2).

One potential explanation for this finding is that the activation of STAT5 along with STAT3 reflects a different cell of origin from tumors with activation of STAT3 alone, and this may underlie the differing biology and outcomes. To address this possibility, a cell line with constitutive activation of STAT3, MDA-MB-468, was transduced with an expression vector for a constitutively activated form of STAT5 or, as a control, the empty vector. The cells with activation of both STAT3 and STAT5 displayed changes in gene expression that paralleled the differences seen in the primary cancers displaying co-activation of STAT3 and STAT5 [19]. Furthermore, the cells with activation of both STAT3 and STAT5 proliferated more slowly, and were more primed to undergo apoptosis as measured by BH3 profiling [21]. These findings suggest that tumors with activation of both STAT3 and STAT5 may also be more susceptible to cell death induced by chemotherapeutic agents such as paclitaxel, which could also contribute to a more favorable prognosis. Furthermore, these results support the hypothesis that the differences in biology between tumors with activation of STAT3 alone or with co-activation of STAT5 are more likely to be driven by these molecular differences rather than based on any potential difference in cell of origin.

*In vitro* studies have shown that STAT3 and STAT5 have essentially identical preferences for binding nine base pair DNA sequences of the form TTCNNNGAA. However, STAT3 and STAT5 mediate disparate biological effects and appear to bind distinct regions in intact chromatin. Gene expression analyses have revealed that some genes are oppositely regulated by STAT3 and STAT5, including the transcriptional repressor BCL6, which can be oncogenic in B cell lymphomas. In breast cancer cells, STAT3 leads to increased expression of BCL6 and STAT5 mediates repression of this gene [19]. When both STAT3 and STAT5 are activated simultaneously, the repressive effect of STAT5 is dominant. This effect of STAT5 is mediated through competition with STAT3 for a common regulatory element within the BCL6 gene [22]. Further studies have revealed that BCL6 is frequently overexpressed in primary breast cancers and breast cancer cell lines, and that BCL6 may play an oncogenic role in breast cancer as well [23]. This suggests that specific gene targets may underlie the distinct biology of tumors with activation of STAT3 alone or with co-activation of STAT5.

Another notable finding from these studies concerns the relative proportion of tumors displaying activation of STAT3 alone *versus* STAT5 alone [19]. While 40% of all breast cancers display activation of STAT3 alone, only 7% of tumors show isolated STAT5 activation. Of the tumors with activated STAT5, more than 80% had activation of STAT3 as well. In addition to promoting survival and proliferation, prolactin also promotes differentiation of mammary epithelium in preparation for lactation. This raises the possibility that isolated activation of STAT5 would not be oncogenic without the coordinate activation of another signaling molecule, such as STAT3, that could prevent tumor differentiation. This finding is also reflected in the fact that very few if any breast cancer cell lines display activation of STAT5 in the absence of exogenous prolactin.

Taken together, these findings indicate that while activated STAT3 can promote malignant cell behavior in breast cancer, important aspects of the biology of tumors characterized by activated STAT3 can be modulated by the presence of activated STAT5. While similar effects have not yet been found in other cancer types, these findings suggest that it is important to understand the status of STAT5 (and perhaps other modifying transcription factors) before one can understand the functional effects of activated STAT3 in a cancer cell.

## 3. STAT3 Target Genes as Modulators of Cancer Pathogenesis: miR-146b

Since the genes regulated by STAT3 control cellular processes such as proliferation, survival, self-renewal, and invasion, it is not surprising that the activation of STAT3 is tightly regulated by several layers of negative feedback regulation. Thus, under physiological conditions, cytokine-induced STAT3 activation reaches maximal levels within 15 to 30 min, and then returns to basal levels within one to two hours. For the constitutive activation of STAT3 to occur, as is seen in cancer, there needs to be activation of some upstream process driving the activity of a kinase that can phosphorylate Tyr-705 of STAT3. However, there likely also needs to be a loss of activity of one or more negative regulators such as phosphatases or SOCS family members, which also occurs commonly in cancer [24,25,26,27]. In addition, STAT3 does not act in isolation, but is affected by other oncogenic transcriptional pathways. For example, the transcription factor NF-κB regulates genes encoding cytokines, such as IL-6, which can trigger the activation of STAT3 [28]. Thus, the status of these feedback loops not only within pathways, but also between pathways, can have a major effect on the biology of a cancer with activated STAT3.

**Figure 3:**
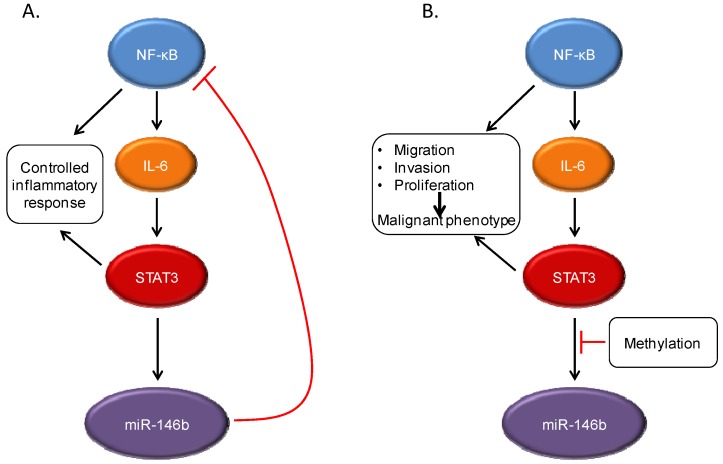
STAT3 interacts with other signaling pathways, including NF-κB. Under normal conditions, NF-κB activation can lead to production of the cytokine IL-6, which can then drive STAT3 activation. However, STAT3 induces expression of the microRNA miR-146b, which then attenuates the activation of NF-κB and decreases IL-6 production. This leads to a controlled inflammatory response (**A**). In cancer cells, the STAT3-induced miR-146b expression does not occur, often through methylation of the miR-146b promoter. This can lead to continued activation of both NF-κB and STAT3, with increased expression of target genes leading to malignant cellular behavior (**B**).

One group of mediators of negative feedback regulation are microRNAs (miRNAs), which can bind to sequences in mRNA and block protein translation and or/promote the degradation of target mRNAs [29]. Although much is known about mRNA targets of STAT3, there has been relatively little published on key miRNA targets of this gene [30,31,32,33]. To identify miRNAs regulated by STAT3 in a biologically relevant system, an activated form of STAT3, STAT3C, was expressed in non-transformed mammary epithelial cells, a system in which STAT3 activation alone is known to be able to mediate oncogenic transformation [34]. Using this approach, with rigorous controls to exclude miRNAs indirectly regulated by STAT3, miR-146b was identified as a direct STAT3 target. Reflecting the overlapping functional effects of STAT family members, miR-146b can also be activated by STAT1 and STAT5. MiR-146b is clearly a physiological STAT target, in that its activation in the mammary gland closely parallels the activation state of STAT3 and STAT5. Notably, however, miR-146b is induced by STAT3 activation only in non-transformed cells. In cancer cell lines, miR-146b was not induced following cytokine-induced STAT3 activation (Figure 3). Furthermore, in primary breast cancers, no increase was seen in miR-146b with increasing STAT3 tyrosine phosphorylation. The hypothesis was considered that epigenetic regulation was preventing STAT3-mediated regulation of this microRNA. In fact, in both cancer cell lines and primary tumors, the miR-146b locus is methylated, thereby silencing its expression. Treatment of cells with the demethylating agent 5-azacytidine restored STAT3-dependent induction of this microRNA. Taken together, these findings raised the question of whether miR-146b was functioning as a tumor suppressor whose loss of expression was selected for during neoplastic progression.

MiR-146b can downregulate components of the NF-κB signaling cascade, including the upstream mediators TRAF6 and IRAK1 [35], and introduction of a synthetic mimic of miR-146b leads to decreased expression of NF-κB target genes. One such NF-κB regulated gene is IL-6, which is produced in an autocrine fashion by many tumor cells and leads to STAT3 phosphorylation via Jak family kinases. Thus, the expression of miR-146b in response to STAT3 activation can directly down-modulate NF-κB signaling, and indirectly decrease the activation of STAT3 itself, in a multi-component feedback system. This can lead to clear biological phenotypes, as ectopic expression of miR-146b in breast cancer cells leads to decreased migration and invasion, and a more epithelial (and less mesenchymal) phenotype. This effect of miR-146b is mechanism-specific, as the introduction of a constitutively active form of STAT3 largely overcomes these effects. This miR-146b-mediated feedback loop between STAT3 and NF-κB is particularly important in that in many systems, chronic inflammation, which leads to NF-κB activation, may be a risk factor for malignant progression [3]. Thus, the ability of miR-146b to downregulate NF-κB activity may be an important homeostatic mechanism in normal tissue function. If miR-146b can no longer be expressed in response to STAT3 activation, as can occur with methylation of this locus, then NF-κB activation would lead to unabated stimulation of STAT3, providing the molecular basis for malignancy.

To determine whether miR-146b acts as a tumor suppressor *in vivo*, primary breast cancers were analyzed for expression of this microRNA. Increased expression of miR-146b was found to be associated with longer survival in patients with tumors that do not express the estrogen receptor, the subtype most likely to display STAT3 activation. This finding suggests that miR-146b may well function in vivo as an endogenous negative feedback regulator with tumor suppressive properties. Thus, once again, the activation state of STAT3 reveals only part of the biology of a tumor. The expression of a key target of STAT3, miR-146b, which can be repressed by DNA methylation, is an important factor in understanding the biology and prognosis of a tumor.

## 4. Conclusions

It is natural to ask whether activated STAT3 is a good or bad prognostic marker in a tumor. However, as we gain more insight into the complexity of the signaling matrix of a cancer cell, it is clear that the answer to that question depends on the status of other signaling and regulatory molecules, including other STATs (such as STAT5) or microRNAs (such as miR-146b). Currently, this ambiguity makes it difficult to answer clinically relevant questions about a patient’s tumor. However, understanding these subtleties reflects our progress in dissecting the signaling events underlying cancer pathogenesis driven by activated STAT3. It also indicates that we are getting closer to appreciating the key biological modulators of STAT3 in a cell, and being able to use this information to design rational molecular therapeutic strategies.
